# Epidemiology and demographics of head and neck cancer in Africa: A scoping review

**DOI:** 10.4102/phcfm.v15i1.3749

**Published:** 2023-08-01

**Authors:** Jaishika Seedat, Kim Coutts, Ellen Vlok

**Affiliations:** 1 Department of Speech and Hearing Therapy, Faculty of Humanities, University of the Witwatersrand, Johannesburg, South Africa

**Keywords:** Africa, head and neck cancer, epidemiology, demographics, scoping review, gender

## Abstract

**Background:**

Low- to middle-income countries account for 70% of global cancer deaths. Evidence of the changing prevalence of head and neck cancer in Africa in terms of gender, race and epidemiology will inform future research and health planning.

**Aim:**

To synthesise epidemiological literature for head and neck cancer in Africa from 2010 to 2020.

**Method:**

A scoping review was completed. The Joanna Briggs Institute Population, context and concept framework confirmed the inclusion criteria. Studies from Africa that included participant demographics, the types, stages, signs and symptoms of head and neck cancer were selected. Five databases were used. Descriptive statistics was completed.

**Results:**

The Preferred Reporting Items for Systematic Reviews and MetaAnalysis guided the reporting of the findings. Of the 1891 articles downloaded, 66 were included in the final review. Nigeria produced the most studies and oral cancer at 74% was most prevalent. Substance abuse was the most prevalent cause. Diagnosis of head and neck cancers were in the late stage (stage IV) when signs and symptoms were severe. Males of lower socioeconomic status tended to have less health seeking behaviour.

**Conclusion:**

Countries from North Africa produce the most research outputs on head and neck cancers. Gender differences were noted and may be linked to lifestyle choices. A range of head and neck cancers (HNCs) are prevalent however late diagnosis and severe symptomatology impact treatment options.

**Contribution:**

Earlier diagnosis and intervention to prevent late-stage diagnosis is necessary. Awareness campaigns linked to evidence on causes, habits and lifestyle choices, signs and symptoms are needed.

## Inroduction

Cancer is the second leading cause of death globally and in 2018 accounted for more than 9.6 million deaths.^1^ The increasing cancer burden globally has an impact on the financial, physical and emotional well-being of individuals, communities and health systems.^2^ In countries that have established health systems, survival rates of various cancer types are improving. This is as a result of accessible and quality detection and treatment.^1^ The countries that do not have a strong health system tend to be countries that are low- to middle-income such as in Africa, where cancer detection and management are untimely and inaccessible.^1^ Despite the increasing prevalence of cancer globally,^2^ research is limited.^3^ In a study conducted in 2018, it was found that Africa accounts for 7.3% of cancer-related deaths.^4^ It is therefore unclear if research on cancer, specifically head and neck cancer (HNC), is reliable, current and identifies a direction for further research.

The current paper therefore presents a scoping review of current research on the epidemiology of HNC in Africa for policymakers, governments within Africa, researchers and healthcare professionals working with HNC by providing evidence for informed decision-making that is context-specific. The first step of decision-making is to understand the epidemiology, in this case that of HNC within Africa, which was the aim of the current study. To understand the significance of epidemiological data of HNC, an understanding of HNC is needed. Head and neck cancer is a vast group of cancers that originate from any area of the head and neck. The predominant anatomical sites originate in the mouth, nose, throat, larynx, sinuses or salivary glands.^5^ Table 1 illustrates the different types of HNCs according to their anatomical structure.

**TABLE 1:** Types of head and neck cancers according to the subsites.^5^

Oral Cancer (OC)	Pharyngeal Cancer (PC)	Laryngeal Cancer (LC)
The mucosal lining of the: Lip Tongue Cheeks Hard palate Floor of the mouth Alveolar ridge Gingiva (gums)	Oropharyngeal cancer (OPC) includes the mucosal lining of the tongue base, palatine tonsils, soft palate and oropharyngeal mucosa and constrictor muscles from the level of the palate to the hyoid bone.	Supraglottis cancer includes the mucosal lining of the epiglottis, aryepiglottic folds, false vocal folds and the deep pre-epiglottic and paraglottic space.
	Nasopharyngeal carcinoma (NPC) includes squamous mucosa (NPSCC), lymphoid tissue (adenoids), the levator palatini muscle and the torus tubarius (the projecting posterior lip of the pharyngeal opening of the eustachian tube).	Glottis cancer includes the squamous mucosa of the vocal cords and thyroid cartilage.
	Hypopharyngeal cancer (HPC) includes the squamous mucosa of: the pyriform sinuses, the pharyngeal walls and inferior and middle constrictors and the postcricoid region.	Subglottis cancer is the mucosal lining of the space between the glottis and trachea and includes the cricoid cartilage.

With reference to Table 1, the distribution of HNC varies across anatomical regions. The most prevalent anatomical regions of the head and neck that are affected by cancer is the upper aerodigestive tract, which includes the oral, pharyngeal and laryngeal structures, but is also inclusive of the upper oesophageal region, that being up to the level of the cervical oesophagus. The primary anatomical structures include the oral, pharyngeal and laryngeal cavities. Each anatomical structure has sub-sites within the cavity that can be affected in isolation or if the HNC has spread to multiple sub-sites.

The following epidemiological data were considered: prevalence, pattern of disease (staging, anatomical sites involved and signs and symptoms experienced), distribution between population groups, lifestyle habits and populations most at risk. To understand the demographics of patients presenting with HNC, age, gender, race and socioeconomic status were considered.

## Methods

A scoping review of the documented epidemiology of HNC in Africa in terms of presentation, types and most common anatomic sites, staging and age at the time of diagnosis as well as information pertaining to gender, race and socioeconomic status as reported in the studies were captured. The data were analysed quantitatively. An ethics waiver was obtained prior to the scoping review commencing, from the university Ethics Committee (Medical) (W-CBP-220218-02). The application for an ethics waiver was in line with the institution’s policy for research involving review (scoping or systematic). Ethical clearance to conduct this study was obtained from the University of the Witwatersrand Human Research Ethics Committee (Medical) (No. R14/49).

### Eligibility criteria as per the Joanna Briggs Institute, population, concept and context framework

*Population*: Patients presenting with head and neck squamous cell carcinoma (HNSCC), all types of nasopharyngeal carcinoma from the nasopharynx to the cervical oesophagus.

*Concept:* Epidemiology, types, stages, signs and symptoms of head and neck cancer, demographics including age, race, gender and socioeconomic status.

*Context*: Africa.

Type of articles included:

- Journal articles.
- Grey literature including unpublished original research (dissertations for degree purposes).
- Published in English.
- Published between January 2010 and June 2020.

### Databases

Embase (via Scopus), Medline (via PubMed, EBSCO or OVID), Web of Science and Google Scholar (the first 200 relevant references) were the search engines used.

### Selection process

A pilot study was conducted to refine the search terms and methods for the main study. Following the pilot study, MeSH terms and controlled vocabulary were not used because of it yielding irrelevant results. Primary keywords used for the main study were ‘head and neck cancer’ and ‘Africa’. After a review of the six databases and 22 grey literature sources, a total of 1891 articles were obtained.

**FIGURE 1:**
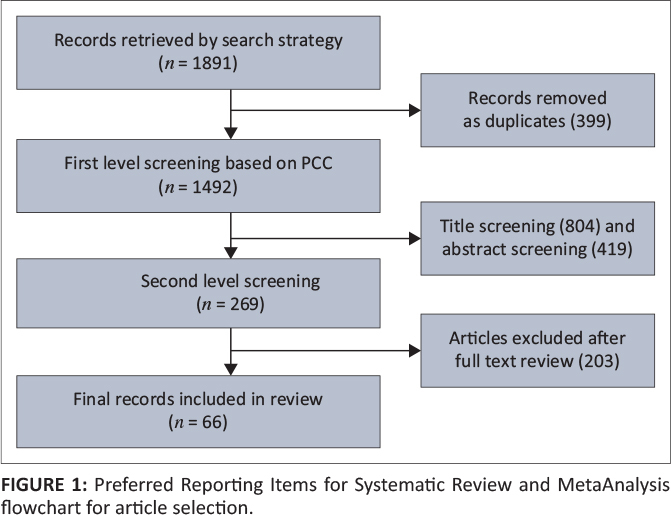
Preferred Reporting Items for Systematic Review and MetaAnalysis flowchart for article selection.

The Cochrane protocol guided the research process.^6^ The data screening process followed the Preferred Reporting Items for Systematic Reviews and MetaAnalyses (PRISMA) flowchart.^7^ This process is described below in Figure 1.

From the 1891 results, 399 duplicates were removed. The first level of screening (title and abstract) removed 1299 articles, leaving 269 articles for the second level of screening. After the second level screening of full articles, 203 articles were removed, leaving *n* = 66 articles in the final review. This is illustrated in Figure 1. Appendix 1 contains details of the studies reviewed.

The data extracted from the reviewed articles followed a data extraction form which was self-developed. The extracted data were appraised according to its quality using the mixed method appraisal tool (MMAT) (Hong et al., 2018) to review the methodological quality of the data obtained from the studies used in the review. The quality of data was high as 68% (*n* = 45) of articles obtained a score of 100% on the MMAT. This can be seen in Appendix 2. All extracted data were synthesised and analysed quantitatively. These data were nominal in nature, and therefore the variables were grouped together, and the frequencies were calculated and compared.

## Review findings

### Epidemiology of head and neck cancer

#### Where were most studies conducted in Africa?

**FIGURE 2:**
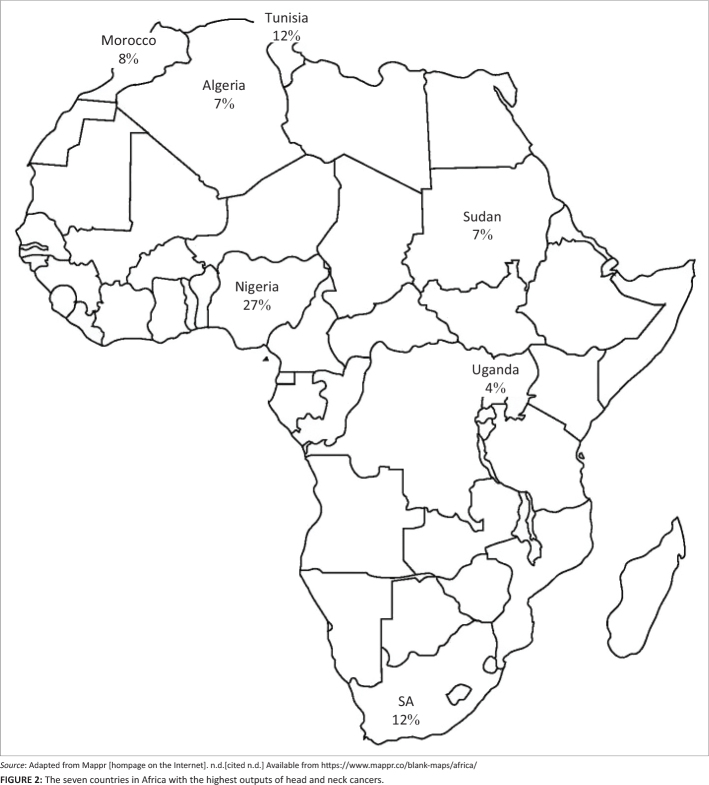
The seven countries in Africa with the highest outputs of head and neck cancers.

Research on HNSCC was conducted in 19 out of the 54 African countries. The seven countries with the highest number of outputs are reflected in Figure 2.^8^

Nigeria (*n* = 20, 27%) conducted most studies with an emphasis on researching oral squamous cell carcinoma (OSCC) or OPSCC and the clinical pattern in different geographical locations within Nigeria. Countries in the Northern portion of Africa tended to study nasopharyngeal carcinoma (NPC). Southern African countries studied the epidemiology of HNSCC, as well as the impact of human immunodeficiency virus (HIV) on HNSCC, as well as human papillomavirus (HPV) as a risk factor for oropharyngeal carcinoma (OPC). Few studies were conducted in eastern and western Africa, with implications for these geographical regions to be explored in future research.

#### Types, anatomic regions and causes of head and neck squamous cell carcinoma

The 66 articles included 35 409 patients diagnosed with HNSCC and/or NPC. Of the 35 409 participants, 35 463 specimens were reported on, as some participants had more than one anatomical region affected. Four types of HNSCC and NPC were identified with the prevalence rates across Africa as follows:

1. HNC unspecified (24 articles) with a prevalence of 14%
2. OC and OPC (23 articles) with a prevalence of 74%
3. NPC (14 articles) with a prevalence of 11%
4. LC/HPC (5 articles) with a prevalence of 1%.

An increase in the prevalence of HNC across Africa was noted resulting from the increase in sexual practices causing HPV, use of tobacco and alcohol. Trends of increasing NPC identified Epstein-Barr virus (EBV), substance abuse and a genetic component as underlying reasons. Oral and oropharyngeal cancers were the most studied HNCs with the highest frequency (74%). Some articles could not identify reasons for the increased prevalence, whilst six articles identified the rise of HPV incidence as a primary cause:^9,10,11,12^

**FIGURE 3:**
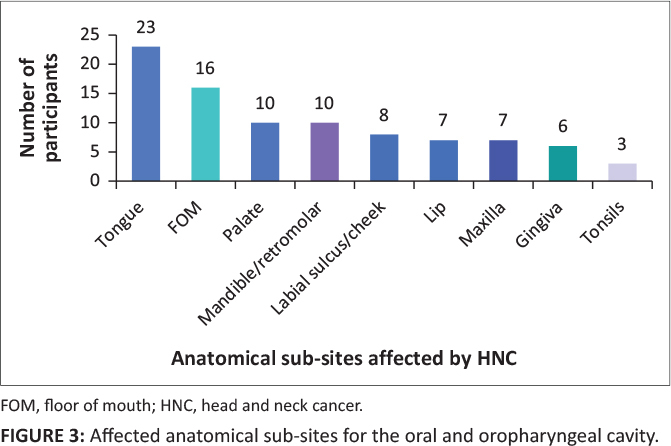
Affected anatomical sub-sites for the oral and oropharyngeal cavity.

1. Most articles in the HNC category did not specify the sub-site, but rather grouped the HNC together. Oral or pharyngeal cancer was the most reported on as it occurred in 16 of the 24 articles and accounted for 1244/2805 of the participants (44%). The rise of OC and OPC is related to the use of tobacco and alcohol and in four countries, to HPV. The countries that reported an increase in HPV as a contributing factor included South Africa, Burkina Faso, Sudan and Ghana.^9,10,11,12^ The results revealed that HPV is strongly related to obtaining OC and OPC, as opposed to other types of HNC. The study done by Ahmed et al.^10^ aligned with this finding as it was stated that ‘… HPV infections were more frequently identified in the tumour tissues from oral (40%) followed by larynx and pharynx’.
2. Figure 3 indicates the articles that researched OC and OPC specifically. The researcher combined these two sub-sites, as many articles reported on these two areas together as they are anatomically proximal to each other.

Most OC and OPC articles did not identify the anatomic subsite but differentiated between OP and OPC (13 out of 23, 57%), of which most participants studied had OPC (71%). There were 10% of articles that did not differentiate a sub-site at all. As noted in Figure 3, the tongue is the most prevalent anatomic subsite (23%). Reasons for this were not specified in the articles. Khammisa et al.^13^ noted that tongue cancer was more prevalent in black people in South Africa:

3. Nasopharyngeal carcinoma was the third most common subcategory found in the articles. The increasing number of NPC was confirmed in that 14 out of 66 (21%) made it the primary focus of the study. On analysis of the HNC subcategory, it was noted that 606 out of 2805 (22%) of the participants with a specified anatomic type had NPC. This is supported by the study done in the southern part of Nigeria (Ibadan)^14^ and found that NPC was the most prevalent of the HNCs. In contrast, the most prevalent of HNCs in southwest Nigeria (Lagos) was of the oral cavity.^15^ Reasons for this difference could not be determined but are interesting to note.

The rise of NPC, particularly in the northern part of Africa, was supported,^16^ where there was an attempt to review the association of gene variants with the susceptibility of HNC (namely NPC) in Tunisia. The results concluded that there are gene variations that cause an increased susceptibility in obtaining NPC, and importantly, there is a ‘… possible role for these variants as biomarkers for early detection of HNC and especially the NPC subtype’.^16^

4. Hypopharyngeal or laryngeal cancer was the fourth category and combined two subsites because of their anatomical proximity. The most reported subsite was the transglottic (48%), followed by the hypopharynx (33%) and the cervical oesophagus (10%). The remaining subsites attributed to 5% or less of the reported sites.

#### Histology of the head and neck squamous cell carcinomas and nasopharyngeal carcinomas

The predominant histological type was squamous cell carcinoma (SCC) (73%), and of this, OC and OPC made up 86% of SCCs. Other (20%) included other carcinomas, lymphomas, sarcomas, benign masses and premalignant masses, as well as articles that did not specify the histological type. Noteworthy *other* cancers included non-Hodgkin lymphoma (*n* = 34/5416, 1%) and Kaposi’s sarcoma (*n* = 27/5416, 1%), even though they make up a small proportion overall.

Nasopharyngeal carcinoma comprised the 7% of the histology reported from all 66 articles. The most prevalent histological type for NPC was WHO type III (UNC), accounting for 97% (*n* = 2238/2317) of the specified NPC histological types. Type I and type II only accounted for 1% (*n* = 16/2317) and 3% (*n* = 63/2317), respectively. The remaining 1804 participants in the NPC subcategory did not have their histological types specified. It is unclear why this was.

#### Stage of head and neck squamous cell carcinoma and nasopharyngeal carcinoma

**FIGURE 4:**
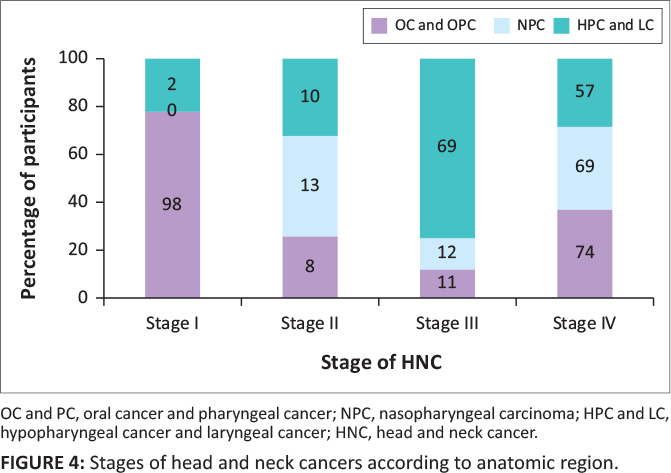
Stages of head and neck cancers according to anatomic region.

In total, there were 19 out of 66 (29%) articles that reported on the stage of HNSCC and NPC, with a total of 2489 participants. Of the articles reporting stage, there were missing data for 16% of the participants (*n* = 404). Furthermore, 5 of the 19 articles only commented on early versus late-stage cancer. Figure 4 shows the articles that commented on the stages and is divided according to the anatomic regions that were affected. The majority of participants (65%) were diagnosed with stage III and stage IV cancer, with 19% presenting with early-stage cancer (stages I and II). Data relating to the stage of HNC were missing for 16% of participants.

With regard to the types of HNC, the largest proportion of stage IV cancers was found in the OC and OPC group (*n* = 272, 74%) and secondly the NPC group (*n* = 400, 69%). Hypopharyngeal cancer and laryngeal cancer was predominantly diagnosed at stage III (*n* = 64, 69%). The HNC with the highest proportion of stage I was OC and OPC (98%) (8%).

#### Signs and symptoms associated with head and neck squamous cell carcinoma and nasopharyngeal carcinoma

**FIGURE 5:**
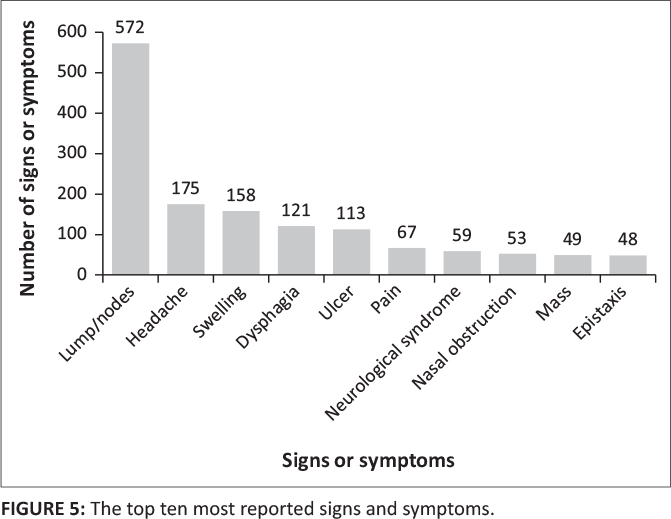
The top ten most reported signs and symptoms.

The distribution of symptoms was widespread. The top 10 most reported signs and symptoms amongst HNC are depicted in Figure 5.

The development of lumps or lymph nodes in the cervical area was, by far, the most reported sign, accounting for 572/2343 (24%) of all reported signs and symptoms. Most articles that reported cranial nerve damage did not specify which cranial nerves were damaged. The largest nasal symptom was nasal obstruction, which accounted for 53 (2%) of all signs and symptoms. The last most reported symptom or sign was epistaxis (nose bleeds) (*n* = 48, 2%).

### Demographic data of head and neck cancer in Africa

#### Gender

A total of 60 out of 66 articles documented the gender of each participant, which included 33 602 participants. Of this, 28% were female (*n* = 9375), and 72% were male (*n* = 24 227). Within the subcategories of OC and OPC, NPC and LCC, all had a higher percentage of males when compared to females. For the OC and OPC subgroup, 25% were females (*n* = 6214), versus 75% being males (*n* = 18 879), which is a ratio of 1:3 (females to males). For the NPC group, there was a predominance of 66% (*n* = 2287) males versus females, accounting for 34% (*n* = 1156), resulting in a ratio of 1:1.9. Finally, for the HPC and LC group, there was 78% males (*n* = 188) to 12% females (*n* = 52).

#### Age

There were 56 articles that documented the mean age of the group, which comprised of 26 818 participants. The overall age mean range was from 19 to 69.9 years, with the largest distribution range being from 36 to 60 years. The largest mean age frequency range is between 56 and 60 years, accounting for 30% of the articles (*n* = 17). There is also a peak noted from 41 to 45 years (*n* = 12) and from 51 to 55 years (*n* = 10). The results indicate a lower overall mean age of people with NPC and then OC and OPC with the highest overall mean age for people with HPC and LC. A potential reason for a lower mean age for the NPV group could be that HIV was prevalent in two of the three studies in Ghana,^17^ Ethiopia^18^ and South Africa,^19^ indicating that people with HIV and NPC presented at a younger age.

#### Socioeconomic status

Socioeconomic status (SES) was discussed in only 11 of the articles, with a total of 1468 participants across the four categories. That included four articles in the HNC category (*n* = 979), four in the OC and OPC group (*n* = 408), two in the NPC group (*n* = 50) and two in the HPC and LC group (*n* = 31). Socioeconomic status was described according to high versus low; level of education, income and/or type of employment for each of the articles. The majority of the participants had a low SES, characterised by a low SES, low education level (primary level or no formal education), low employment level (labourers, peasants, artisans; unemployed or students) or a low-income level. Low income was defined by each article, according to the national breadline, which included earning less or more than R688.00 and R3000.00 in Uganda and Nigeria, respectively. Both Nigeria and Uganda used their own currency; however, this was converted into South African Rands for ease of comparison. Education level was the only SES marker to be greater in the high SES group (54.87%), compared to the low SES group (45.13%). The higher percentage is because secondary level education was included in the high-level group, which accounted for 68% of the high group. Only 32% of the participants had a high level of education or tertiary level of education.

Furthermore, it was found that those of a lower SES tended to have more advanced stage HNC, and a potential reason for this was ‘… because of their inability to afford treatment, ignorance, incorrect diagnosis and reliance on native medications’.^20^ This is in keeping with the primary theme of poor access to healthcare and resources, suggesting that those of a lower SES are at a greater disadvantage.

There was no significant difference of SES between the types of HNC noted. No comparison between regions could be made, as SES was demonstrated differently between countries. Furthermore, limited associations could be made between SES and the risk factors, as the results within the articles did not include associations, and there was no access to the raw data.

#### Race

Race was mentioned in eight studies, comprising of 24 462 participants. Three of the eight studies were case reports of single participants. There were six that were conducted in South Africa, of which three were done in the city of Johannesburg. The other two were conducted in Ethiopia (*n* = 1) and Malawi (*n* = 1). Of the articles mentioning race, seven were in the OC and OCP subcategory and one case study for NPC. Data pertaining to race were missing for 407 participants. A predominance of black people is shown, accounting for 56% (*n* = 13 355) of the participants. There were two studies^21,22^ that investigated the race with the highest prevalence when compared to the population distribution. It was found in both studies that mixed race males are the most prevalent race with OC and OPC in South Africa and are therefore most at risk, even though the mixed race population only accounted for 12% of the participants (*n* = 2984). Possible reasons for an increased risk within the mixed race population were not conclusive, although both articles suggested an increase in smoking, in conjunction with high alcohol use.^21,22^

## Discussion

With only 66 articles included in the scoping review, there is a clear dearth of information in the field of epidemiology of HNC in Africa. Data reflected findings from 19 of the 54 African countries. Literature concedes that there is limited clinical research across Africa generally, with only 1% of the world’s research being done in Africa, even though it accounts for 12% of the world’s population.^23^

A high number of full-text articles were excluded from the study (*n* = 203), as they did not specifically study HNSCC or NPC (*n* = 99). Many articles did not answer the objectives of the study (*n* = 63), whilst 16 articles were not the primary source of information.

Head and neck cancer is on the rise in low- to middle-income countries, whilst some developed countries have declining numbers, particularly of OC.^24^ Simard et al.^24^ reviewed HNC across five continents excluding Africa, showing declining OC incidence in Asian countries, Canada and the United States. Oropharyngeal cancer rates, however, increased in some European countries but decreased in Asian countries. The rise of HPV was a contributory factor.^25^ The rise of HNC is similar to Africa, even though overall prevalence was not documented; however, this has been confirmed in other studies.^26^ As a result, prioritising HNC as a field of research, policymaking and education programmes is indicated.

The most studied and reported sub-site of HNC in this review was OC and OPC. Results showed that OPC was reported more than OC. This correlates with international findings where OC is higher in countries where tobacco is still a large risk factor (Slovak Republic, Estonia, Finland and Japan) and OPC being high in countries with decreasing tobacco use trends, but increasing HPV (Belarus, Czech Republic, Denmark, Finland, Iceland, Latvia, Norway and the United Kingdom).^25^

In terms of histology, SCCs were noteworthy for being the most common globally and in Africa.^27^ The histology of SCC is aggressive and fast spreading. In countries such as the United States and the United Kingdom, the few cases of NPCs are typically SCC in nature.^28^ In recent years, however, NPC type III is of epidemic standards in Southeast Asia, Southern China and North African countries and is largely associated with EBV.^27^ The systematic review supports this notion, as most NPCs were found in Northern Africa, as well as some northeast African countries (Sudan) and are mostly type III in histology. Nasopharyngeal carcinoma in Northern Africa is typically associated with a comorbidity (unspecified).^29^ The systematic review also supports a worse outcome with the later stage diagnosis.

Late-stage HNC was common in the studies in Africa. Interestingly both developed and developing have late-stage diagnoses.^30,31,32^ This is likely related to lifestyle habits in those of a lower SES and in males, regardless of country. Individuals from a lower SES and males tend to have less health-seeking behaviours and are diagnosed at a later stage.^33,34^ The current review concurred. Importantly, HNCs tend to have a high metastatic rate and are aggressive in nature and can be difficult to diagnose as they mimic a common cold or flu^5^ such as swollen lymph nodes, headaches, pain in the affected area (nose, mouth or throat), coughing, ulcer development, loss of taste or smell, nasal obstruction or congestion and nasal discharge.^35^

Males over 40 years and from a lower SES are most at risk in Africa. A South African study^36^ revealed that 70.4% of those diagnosed with HNC were black people (that being 1131 out of 1605 files). This aligned with international studies that found that tobacco and alcohol use by males versus females contributed to this.^4^

The highest proportion of females found in the systematic review was in the NPC group, accounting for 34% of the NPCs. The higher number of females and higher proportion of NPC in women in Africa, versus other HNCs was not explained in the articles. However, it is likely that the association of EBV and a genetic predisposition causing females to have NPC is possible, but this warrants further research for confirmation. The OC and OPC group had a higher proportion of women when compared to other HNCs, and when compared to OC and OPC in previous years because of the growing rate of HPV. The effect of HIV on gender differences for HNC still needs exploring.

The average age of HNC effects was from 56 to 60 years, similar to the study from South Africa.^36^ This is slightly less than the average age in developed countries, for example, the United States with an average age range of 50–70 years.^37^ Age seems to not be a significant demographic factor for developing OC and OPC in Africa. Rather, HPV and HIV have an impact on the age of OC and OPC diagnoses, of which Africa is at a high risk.^1,38^

Most people with HNC had a low SES, characterised by low income, being unemployed or labourers and/or having no formal education or only primary education. No associations could be made between the SES to various regions and to the associated risk factors, and this needs to be further explored in Africa.

Race was documented in South Africa only. Africa in comparison used ethnicity differences. Literature concedes that HNC is not particularly race related, but rather depends on social and cultural practices within different ethnic groups.^39^

## Conclusion

Nigeria conducted the most studies on HNC in Africa. Oral squamous cell carcinomas or oropharyngeal squamous cell carcinomas were the most prevalent types of HNC, with a rise of nasopharyngeal carcinoma (type III) in Northern Africa. Diagnosis was late (stage IV). Substance abuse, namely tobacco use, was the most prevalent cause, with a rise of HPV-related HNC and EBV-related nasopharyngeal cancer across Africa. Males and those of lower SES were most at risk.

This scoping review has limitations. The first is the limitation to the data obtained. No raw data were used; therefore, it is a summary and analysis of other researcher’s data collection. Because of missing data in some of the articles, sample sizes were small. Causal relationships were therefore difficult to make. Another challenge was the various foci across the epidemiological studies, across a large space. As a result, it was difficult to generalise findings, as each country had a unique epidemiological pattern. This needs to be further explored in each country.

## Appendix 1

**TABLE 1-A1:** Description of articles reviewed.

Authors	Title of article	Country of research	Data collection period
Aboagye et al., 2019	Human papillomavirus detection in head and neck squamous cell carcinomas at a tertiary hospital in Sub-Saharan Africa	Ghana	2007–2016
Abram et al., 2012	Epidemiology of oral squamous cell carcinoma.	South Africa	1997–2009
Adesina et al., 2018	Review of 109 cases of primary malignant orofacial lesions seen at a Nigerian tertiary hospital	Nigeria	2008–2017
Adewuyi et al., 2013	Clinicopathologic characterization of nasopharyngeal carcinoma seen in the radiotherapy and oncology department, ahmadu bello university teaching hospital, Zaria, Nigeria: 2006–2010	Nigeria	2006–2010
Adeyemi et al., 2013	Clinical presentation of oral squamous cell carcinoma	Nigeria	1990–2008
Adeyemi et al., 2011	A retrospective histopathological review of oral squamous cell carcinoma in a Nigerian teaching hospital.	Nigeria	1990–2008
Adeyemi et al., 2011	Oral squamous cell carcinoma, socioeconomic status and history of exposure to alcohol and tobacco	Nigeria	1990–2008
Adoga et al., 2010	Clinicopathological profile of malignant tumors of the oropharynx: a case series.	Nigeria	1998–2008
Ahmed et al., 2012	Human papilloma virus attributable head and neck cancer in the Sudan assessed by p16ink4a immunostaining	Sudan	-
Akinshipo et al., 2017	Head and neck cancers: An histopathologic review of cases seen in three tertiary hospitals in North-western Nigeria	Nigeria	2006–2013
Alabi et al., 2010	Clinico-pathological pattern of nasopharyngeal carcinoma in lorin, Nigeria	Nigeria	1999–2008
Alex-Okoro et al., 2016	The comparison of the pathological data of oropharyngeal masses between HIV and non-HIV patients	Nigeria	2007–2014
Amusa et al., 2011	Laryngeal carcinoma: Experience in lle-lfe, Nigeria	Nigeria	1994–2004
Asante et al., 2017	Detection of human papillomavirus genotypes and epstein-barr virus in nasopharyngeal carcinomas at the korle-bu teaching hospital, Ghana	Ghana	2006–2012
Ayo-Yusuf et al., 2013	Trends and ethnic disparities in oral and oro-pharyngeal cancers in South Africa, 1992–2001.	South Africa	1992–2001
Bassey et al., 2015	Analysis of 46 cases of malignant jaw tumours in Calabar, Nigeria	Nigeria	2000–2013
Blumberg et al., 2015	Investigation of the presence of HPV related oropharyngeal and oral tongue squamous cell carcinoma in Mozambique.	Mozambique	2005–2013
Butt et al., 2012	Oral squamous cell carcinoma in human immunodeficiency virus positive patients: Clinicopathological audit	Kenya	-
dos Passos et al., 2015	Loupe magnification for head and neck free flap reconstruction in a developing country	South Africa	-
Douthit et al., 2016	Social determinants of health: Poverty, national infrastructure and investment	Ethiopia	-
Edreis et al., 2016	Molecular detection of epstein – barr virus in nasopharyngeal carcinoma among sudanese population	Sudan	2015–2016
El–Amrani-Joutey et al., 2018	Infection by Epstein–Barr virus in Fes (Morocco). Prevalence and predictors of positivity in nasopharyngeal cancer	Morocco	2012–2014
Elfeky et al., 2015	Hypopharyngeal reconstruction: a comparison of three alternatives	Egypt	2007–2010
Erasmus et al., 2013	The histology of nasopharyngeal masses: A comparison between HIV positive and HIV negative patients	South Africa	2006–2011
Erinoso et al., 2016	Emerging trends in the epidemiological pattern of head and neck cancers in Lagos, Nigeria	Nigeria	2003–2013
Faggons et al., 2017	Human papilloma virus in head and neck squamous cell carcinoma: A descriptive study of histologically confirmed cases at Kamuzu Central Hospital in Lilongwe, Malawi	Malawi	2010–2014
Garrana et al., 2018	Oral Squamous Cell Carcinoma, a growing problem.	South Africa	1990–2010
Gilyoma et al., 2015	Head and neck cancers: A clinico-pathological profile and management challenges in a resource-limited setting	Tanzania	2009–2013
Hounkpatin et al., 2020	Histo-epidemiological profile of head and neck cancers in benin	Benin	2009–2014
Ilboudo et al., 2019	Characterization of high-risk oncogenic human papillomavirus genotypes in histologically confirmed ear, nose and throat (Ent) cancers in burkina faso	Burkina Faso	2007–2017
Iseh et al., 2011	Total laryngectomy for laryngeal cancer in a Nigerian tertiary health center: Prognosis and outcome	Nigeria	2000–2009
Jalouli et al., 2010	Presence of human papilloma virus, herpes simplex virus and Epstein-Barr virus DNA in oral biopsies from Sudanese patients with regard to toombak use	Sudan	-
Jalouli et al., 2012	Human papilloma virus, herpes simplex virus and Epstein Barr virus in oral squamous cell carcinoma from eight different countries	Sudan	-
Kakande et al., 2010	Head and neck squamous cell carcinoma in a Ugandan population: A descriptive epidemiological study	Uganda	2004–2009
Kamulegey et al., 2017	Head and neck cancers case control study of HIV positive compared to negative patients in a Ugandan population sample	Uganda	2014–2017
Kariche et al., 2018	Comparative assessment of HPV, alcohol and tobacco etiological fractions in Algerian patients with laryngeal squamous cell carcinoma	Algeria	2012–2016
Khaali et al., 2016	No association between TGF-β1 polymorphisms and risk of nasopharyngeal carcinoma in a large North African case-control study	Algeria, Morocco, Tunisia	2001–2004
Khammissa et al., 2014	Oral squamous cell carcinoma in a South African sample: Race or ethnicity, age, gender, and degree of histopathological differentiation	South Africa	1995–2002
Khlifi et al., 2014	Risk of laryngeal and nasopharyngeal cancer associated with arsenic and cadmium in the Tunisian population	Tunisia	2007–2009
Khlifi et al., 2013	Arsenic, cadmium, chromium and nickel in cancerous and healthy tissues from patients with head and neck cancer	Tunisia	2007–2009
Kodiya et al., 2016	Epidemiology of head and neck cancers in Maiduguri-Northeastern Nigeria	Nigeria	2010–2014
Kofi et al., 2019	Infrequent detection of human papillomavirus infection in head and neck cancers in the Central African Republic: A retrospective study	Central African Republic	2009–2017
Laantri et al., 2011	XRCC1 and hOGG1 genes and risk of nasopharyngeal carcinoma in North African countries	Algeria, Morocco, Tunisia	2001–2004
Lasisi et al., 2012	Oro-facial squamous cell carcinoma – A twenty-year retrospective clinicopathological study.	Nigeria	1990–2009
Lawal et al., 2011	Social profile and habits of oral cancer patients in Ibadan.	Nigeria	1 year, 6 months
Makni et al., 2019	Association of common il-10 promoter gene variants with the susceptibility to head and neck cancer in Tunisia	Tunisia	2012–2015
Masamba et al., 2013	Case Report: Down-staging locally advanced head and neck cancer in an HIV infected patient in a limited resource setting	Malawi	-
Milad et al., 2018	Prevalence of human papillomavirus in benign and malignant laryngeal lesions in Egyptian patients: Cross-sectional study	Egypt	2014–2015
Mokni-Baizig et al., 2017	HLA-A*26-A*30 and HLA-DRB1*10 could be predictors of nasopharyngeal carcinoma risk in high-risk Tunisian families	Tunisia	-
Molomo et al., 2015	Discoid lupus erythematosus-related squamous cell carcinoma of the lip in an HIV-seropositive black male	South Africa	-
Moumad et al., 2018	Joint effect of smoking and NQO1 C609T polymorphism on undifferentiated nasopharyngeal carcinoma risk in a North African population	Algeria, Morocco, Tunisia	2002–2004
Moumad et al., 2013	Genetic polymorphisms in host innate immune sensor genes and the risk of nasopharyngeal carcinoma in North Africa	Algeria, Morocco, Tunisia	2001–2004
Mwansasu et al., 2015	Pattern of head and neck cancers among patients attending muhimbili national hospital Tanzania	Tanzania	2012–2013
Nabukenya et al., 2018	Head and neck squamous cell carcinoma in western Uganda: Disease of uncertainty and poor prognosis	Uganda	2016–2016
Ndiaye et al., 2013	The role of human papillomavirus in head and neck cancer in Senegal	Senegal	2002–2010
Oga et al., 2016	Paucity of HPV-related head and neck cancers (HNC) in Nigeria	Nigeria	1990–2011
Okwor et al., 2017	Survivorship of patients with head and neck cancer receiving care in a tertiary health facility in Nigeria	Nigeria	2002–2011
Omitola et al., 2017	A multi-centre evaluation of oral cancer in southern and western Nigeria: An African oral pathology research consortium initiative	Nigeria	1990–2016
Osman et al., 2010	Pattern of malignant tumors registered at a referral oral and maxillofacial hospital in Sudan during 2006 and 2007	Sudan	2006–2007
Paquette et al., 2013	Evidence that alpha-9 human papillomavirus infections are a major etiologic factor for oropharyngeal carcinoma in black South Africans	South Africa	2005–2010
Raissouni et al., 2013	Clinical prognostic factors in locally advanced nasopharyngeal carcinoma in Moroccan population	Morocco	2003–2005
Rettig et al., 2019	Oral human papillomavirus infection and head and neck squamous cell carcinoma in rural northwest cameroon	Cameroon	2011–2017
Sabageh et al., 2015	Malignant tumors of the upper aerodigestive tract as seen in a Nigerian tertiary health institution	Nigeria	2000–2009
Sekee et al., 2018	Human papillomavirus in head and neck squamous cell carcinomas in a South African cohort	South Africa	2014–2017
Tealab et al., 2019	Prevalence of human papilloma virus in oropharyngeal, tongue and lip squamous cell carcinoma: An experience from the Egyptian national cancer institute	Egypt	2008–2015
Wided et al., 2015	Nasopharyngeal carcinoma incidence in North Tunisia: Negative trends in adults but not adolescents, 1994–2006	Tunisia	1994–2006

A dash is in place where the date was not specified.

## Appendix 2: Mixed Methods Appraisal Tool (MMAT)

Responses for each:

- ‘Yes’ response: Provide 20%
- ‘No; or ‘can’t tell’ response: Provide 0

### Mixed Methods Appraisal Tool criteria for qualitative studies:

1. Is the qualitative approach appropriate to answer the research question?
2. Are the qualitative data collection methods adequate to address the research question?
3. Are the findings adequately derived from the data?
4. Is the interpretation of results sufficiently substantiated by data?
5. Is there coherence between qualitative data sources, collection, analysis and interpretation?

**TABLE 1-A2:** Mixed method appraisal tool for qualitative studies included in the scoping review.

Authors	Article title	Q1	Q2	Q3	Q4	Q5	Score (%)	Comment
Douthit et al., 2016	Social determinants of health: Poverty, national infrastructure and investment	Yes	Yes	Yes	Yes	Yes	100	N/A
Masamba et al., 2013	Case Report: Down-staging locally advanced head and neck cancer in an HIV infected patient in a limited resource setting	Yes	Yes	Yes	Yes	Yes	100	N/A
Molomo et al., 2015	Discoid lupus erythematosus-related squamous cell carcinoma of the lip in an HIV-seropositive black male	Yes	Yes	Yes	Yes	Yes	100	N/A

### Mixed Methods Appraisal Tool criteria for quantitative nonrandomised studies:

1. Are the participants representative of the target population?
2. Are measurements appropriate regarding both the outcome and intervention (or exposure)?
3. Are there complete outcome data?
4. Are the confounders accounted for in the design and analysis? (In explanation in MMAT: Confounders are factors that predict both the outcome of interest and the intervention received/exposure at baseline)
5. During the study period, is the intervention administered (or exposure occurred) as intended?

**TABLE 2-A2:** Mixed methods appraisal tool for quantitative nonrandomised studies included in the scoping review.

Authors	Article title	Q1	Q2	Q3	Q4	Q5	Score (%)	Comment
**HNC sub-group**								
Aboagye et al., 2019	Human papillomavirus detection in head and neck squamous cell carcinomas at a tertiary hospital in Sub-Saharan Africa	Yes	Yes	Yes	Yes	Yes	100	N/A
Ahmed et al., 2012	Human papilloma virus attributable head and neck cancer in the Sudan assessed by p16ink4a immunostaining	Yes	Yes	Yes	Yes	Yes	100	N/A
Faggons et al., 2017	Human papilloma virus in head and neck squamous cell carcinoma: A descriptive study of histologically confirmed cases at kamuzu central hospital in Lilongwe, Malawi	Yes	Yes	Yes	No	Yes	80	Article does not state any other risk factors that the participants could have been exposed to prior to or in conjunction with the studied risk factor. This could not be concluded as the article mentioned that other risk factors may have been included, but did not specify numeric values.
Ilboudo et al., 2019	Characterization of high-risk oncogenic human papillomavirus genotypes in histologically confirmed ear, nose and throat (Ent) cancers in Burkina Faso	Yes	No	Yes	No	Yes	60	Did not measure as intended, as missing data, and Incorrect documented values. Therefore, confounders could not be accounted for, and measurements not appropriate/correct.
Kamulegey et al., 2017	Head and neck cancers case control study of HIV positive compared to negative patients in a Ugandan population sample	Yes	Yes	Yes	Yes	Yes	100	N/A
Khlifi et al., 2014	Risk of laryngeal and nasopharyngeal cancer associated with arsenic and cadmium in the Tunisian population	Yes	Yes	Yes	Yes	Yes	100	N/A
Khlifi et al., 2013	Arsenic, cadmium, chromium and nickel in cancerous and healthy tissues from patients with head and neck cancer	Yes	Yes	Yes	Yes	Yes	100	N/A
Kofi et al., 2019	Infrequent detection of human papillomavirus infection in head and neck cancers in the Central African Republic: A retrospective study	Yes	Yes	Yes	Yes	Yes	100	N/A
Makni et al., 2019	Association of common il-10 promoter gene variants with the susceptibility to head and neck cancer in Tunisia	Yes	Yes	Yes	Yes	Yes	100	N/A
Ndiaye et al., 2013	The role of human papillomavirus in head and neck cancer in Senegal	Yes	Yes	Yes	Yes	Yes	100	N/A
Oga et al., 2016	Paucity of HPV-related head and neck cancers (HNC) in Nigeria	No	Yes	No	Yes	Cannot tell	40	Small sample, missing data, and incorrect clinical records. Therefore, confounders could not be accounted for, and measurements not appropriate/correct. Not representative of Nigerian population.
Rettig et al., 2019	Oral human papillomavirus infection and head and neck squamous cell carcinoma in Rural Northwest Cameroon	No	Yes	Yes	Yes	Yes	80	Not representative of target population, as study only completed at one tertiary hospital.
Sekee et al., 2018	Human papillomavirus in head and neck squamous cell carcinomas in a South African cohort	Cannot tell	Yes	Yes	Yes	Yes	80	Did not state target population within South Africa.
**Oral and oropharyngeal SCC subgroup**								
Alex-Okoro et al., 2016	The comparison of the pathological data of oropharyngeal masses between HIV and non-HIV patients	Yes	Yes	Yes	Yes	Yes	100	N/A
Blumberg et al., 2015	Investigation of the presence of HPV related oropharyngeal and oral tongue squamous cell carcinoma in Mozambique	Yes	Yes	Yes	Yes	Yes	100	N/A
Jalouli et al., 2010	Presence of human papilloma virus, herpes simplex virus and Epstein-Barr virus DNA in oral biopsies from Sudanese patients with regard to toombak use	Yes	Yes	Yes	Yes	Yes	100	N/A
Jalouli et al., 2012	Human papilloma virus, herpes simplex virus and Epstein Barr virus in oral squamous cell carcinoma from eight different countries	Yes	Yes	Yes	Yes	Yes	100	N/A
Lawal et al., 2011	Social profile and habits of oral cancer patients in Ibadan	Yes	Yes	Yes	Yes	Yes	100	N/A
Paquette et al., 2013	Evidence That Alpha-9 human papillomavirus infections are a major etiologic factor for oropharyngeal carcinoma in Black South Africans	Yes	Yes	Yes	Yes	Yes	100	N/A
Tealab et al., 2013	Prevalence of human papilloma virus in oropharyngeal, tongue and lip squamous cell carcinoma: An experience from the Egyptian National Cancer Institute	Yes	Yes	Yes	Yes	Yes	100	N/A
**NPC subgroup**								
Asante et al., 2017	Detection of human papillomavirus genotypes and epstein-barr virus in nasopharyngeal carcinomas at the korle-bu teaching hospital, Ghana	Yes	Yes	Yes	Cannot tell	Yes	80	Did not discuss potential confounders in article therefore do not know if accounted for.
Edreis et al., 2016	Molecular detection of epstein – barr virus in nasopharyngeal carcinoma among sudanese population	Cannot tell	Yes	Yes	Yes	Yes	80	Did not state target population if for representation of entire Sudanese population, or within particular area.
El-Amrani-Joutey et al., 2018	Infection by Epstein–barr virus in fes (Morocco). Prevalence and predictors of positivity in nasopharyngeal cancer	Yes	Yes	Yes	Yes	Yes	100	N/A
Erasmus et al., 2013	The histology of nasopharyngeal masses: A comparison between HIV positive and HIV negative patients	Cannot tell	Yes	Yes	Yes	Yes	80	Did not state target population – only HIV positive and negative. Cannot tell if targeted.
Khaali et al., 2016	No association between TGF-β1 polymorphisms and risk of nasopharyngeal carcinoma in a large North African case-control study	Yes	Yes	Yes	Yes	Yes	100	N/A
Laantri et al., 2011	XRCC1 and hOGG1 genes and risk of nasopharyngeal carcinoma in North African countries	Yes	Yes	Yes	Yes	Yes	100	N/A
Mokni-Baizig et al., 2013	HLA-A*26-A*30 and HLA-DRB1*10 could be predictors of nasopharyngeal carcinoma risk in high-risk Tunisian families	Yes	Yes	Yes	Yes	Yes	100	N/A
Moumad et al., 2018	Joint effect of smoking and NQO1 C609T polymorphism on undifferentiated nasopharyngeal carcinoma risk in a North African population	Yes	Yes	Yes	Yes	Yes	100	N/A
Moumad et al., 2013	Genetic polymorphisms in host innate immune sensor genes and the risk of nasopharyngeal carcinoma in North Africa	Yes	Yes	Yes	Yes	Yes	100	N/A
Raissouni et al., 2013	Clinical prognostic factors in locally advanced nasopharyngeal carcinoma in Moroccan population	Yes	Yes	Cannot tell	Yes	No	60	Small sample, missing data, and incorrect clinical records. Therefore, confounders could not be accounted for, and measurements not appropriate or correct.
**Laryngeal and hypopharyngeal SCC**								
Kariche et al., 2018	Comparative assessment of HPV, alcohol and tobacco etiological fractions in Algerian patients with laryngeal squamous cell carcinoma	Yes	Yes	Yes	Yes	Yes	100	N/A
Milad et al., 2018	Prevalence of human papillomavirus in benign and malignant laryngeal lesions in Egyptian patients: Cross-sectional study	Yes	Yes	Yes	Yes	Yes	100	N/A

HNC, head and neck cancer; SCC, squamous cell carcinoma; NPC, nasopharyngeal carcinoma; HPV, human papillomavirus; N/A, not applicable.

### Mixed Methods Appraisal Tool criteria for quantitative descriptive studies:

1. Is the sampling strategy relevant to address the research question?
2. Is the sample representative of the target population?
3. Are the measurements appropriate?
4. Is the risk of nonresponse bias low?

Explanation: Nonresponse bias consists of ‘an error of non-observation reflecting an unsuccessful attempt to obtain the desired information from an eligible unit’ (Federal Committee on Statistical Methodology, 2001, p. 6). To judge this criterion, consider whether the respondents and nonrespondents are different on the variable of interest. This information might not always be reported in a paper. Some indicators of low nonresponse bias can be considered such as a low nonresponse rate, reasons for nonresponse (e.g. noncontacts vs. refusals) and statistical compensation for nonresponse (e.g. imputation). The nonresponse bias might not be pertinent for case series and case report. This criterion could be adapted. For instance, complete data on the cases might be important to consider in these designs.

5. Is the statistical analysis appropriate to answer the research question?

**TABLE 3-A2:** Mixed method appraisal tool for quantitative studies included in the scoping review.

Author	Document title	Q1	Q2	Q3	Q4	Q5	Score (%)	Comment
**HNC subgroup**								
Akinshipo et al., 2017	Head and neck cancers: An histopathologic review of cases seen in three tertiary hospitals in Northwestern Nigeria	Yes	Yes	Yes	Yes	Yes	100	N/A
Dos Passos et al., 2015	Loupe magnification for head and neck free flap reconstruction in a developing country	Yes	Yes	Yes	Yes	Yes	100	N/A
Erinoso et al., 2016	Emerging trends in the epidemiological pattern of head and neck cancers in Lagos, Nigeria	Yes	Yes	Yes	Yes	Yes	100	N/A
Gilyoma et al., 2015	Head and neck cancers: A clinico-pathological profile and management challenges in a resource-limited setting	Yes	Yes	Yes	No	Yes	80	Nonresponse bias high. Small sample, and did not indicate the number of people that were excluded or did not participate in the study.
Hounkpatin et al., 2020	Histo-Epidemiological Profile of Head and Neck Cancers in Benin	Yes	Yes	Yes	Yes	Yes	100	N/A
Kakande et al., 2010	Head and neck squamous cell carcinoma in a Ugandan population: A descriptive epidemiological study	Yes	Yes	Yes	Cannot tell	Yes	80	Nonresponse bias not low. Small sample, and did not indicate the number of people that were excluded or did not participate in the study.
Kodiya et al., 2015	Epidemiology of head and neck cancers in maiduguri-Northeastern Nigeria	Yes	No	Yes	Cannot tell	Yes	60	Sample not representative, as small sample size. Nonresponse bias not low. Did not indicate the number of people that were excluded or did not participate in the study.
Mwansasu et al., 2015	Pattern of head and neck cancers among patients attending muhimbili national hospital Tanzania	Yes	Yes	Yes	Cannot tell	Yes	80	Nonresponse bias not low. Small sample, and did not indicate the number of people that were excluded or did not participate in the study.
Nabukenya et al., 2018	Head and neck squamous cell carcinoma in Western Uganda: Disease of uncertainty and poor prognosis	Yes	Yes	Yes	Yes	Yes	100	N/A
Okwor et al., 2017	Survivorship of patients with head and neck cancer receiving care in a tertiary health facility in Nigeria	Yes	Yes	Yes	Yes	Yes	100	N/A
Sabageh et al., 2015	Malignant tumors of the upper aerodigestive tract as seen in a Nigerian tertiary health institution	Yes	Cannot tell	Yes	Yes	Yes	80	Did not mention target population
**Oral and oropharyngeal SCC subgroup**								
Abram et al., 2012	Epidemiology of oral squamous cell carcinoma	Yes	Cannot tell	Yes	Yes	Yes	100	Did not mention target population
Adesina et al., 2018	Review of 109 cases of primary malignant orofacial lesions seen at a Nigerian tertiary hospital	Yes	Yes	Yes	Yes	Yes	100	N/A
Adeyemi et al., 2013	Clinical presentation of oral squamous cell carcinoma	Yes	Yes	Yes	Cannot tell	No	60	Reported missing data and small sample size, however, did not adjust statistics to this. Also unable to tell if accounted for risk bias, as did not indicate the number of people that were excluded or did not participate in the study.
Adeyemi et al., 2011a	A retrospective histopathological review of oral squamous cell carcinoma in a Nigerian teaching hospital	Yes	Yes	Yes	Cannot tell	Yes	80	Unable to tell if accounted for risk bias, as did not indicate the number of people that were excluded or did not participate in the study
Adeyemi et al., 2011b	Oral squamous cell carcinoma, socioeconomic status and history of exposure to alcohol and tobacco	Yes	Yes	Yes	Cannot tell	Yes	80	Unable to tell if accounted for risk bias, as did not indicate the number of people that were excluded or did not participate in the study
Adoga et al., 2010	Clinicopathological profile of malignant tumors of the oropharynx: A case series	Yes	Yes	Yes	Yes	Yes	100	N/A
Ayo-Yusuf et al., 2013	Trends and ethnic disparities in oral and oro-pharyngeal cancers in South Africa, 1992–2001	Yes	Yes	Yes	Cannot tell	Yes	80	Unable to tell if accounted for risk bias, as did not indicate the number of people that were excluded or did not participate in the study
Bassey et al., 2015	Trends and ethnic disparities in oral and oro-pharyngeal cancers in South Africa, 1992–2001	Yes	Yes	Yes	Yes	Yes	100	N/A
Butt et al., 2015	Oral squamous cell carcinoma in human immunodeficiency virus positive patients: Clinicopathological audit	Yes	Yes	Yes	Cannot tell	Yes	80	Unable to tell if accounted for risk bias, as did not indicate the number of people that were excluded or did not participate in the study
Garrana et al., 2018	Oral Squamous Cell Carcinoma, a growing problem	Yes	Yes	Yes	Yes	Yes	100	N/A
Khammissa et al., 2014	Oral squamous cell carcinoma in a South African sample: Race/ethnicity, age, gender, and degree of histopathological differentiation	Yes	Yes	Yes	Yes	Yes	100	N/A
Lasisi et al., 2012	Oro-facial squamous cell carcinoma – A twenty-year retrospective clinicopathological study	Yes	Yes	Yes	Cannot tell	Yes	80	Unable to tell if accounted for risk bias, as did not indicate the number of people that were excluded or did not participate in the study
Omitola et al., 2017	A multi-centre evaluation of oral cancer in southern and Western Nigeria: An African oral pathology research consortium initiative	Yes	Yes	Yes	Yes	Yes	100	N/A
Osman et al., 2010	Pattern of malignant tumors registered at a referral oral and maxillofacial hospital in Sudan during 2006 and 2007	Yes	Yes	Yes	Yes	Yes	100	N/A
**NPC subgroup**								
Adewuyi et al., 2013	Clinicopathologic characterization of nasopharyngeal carcinoma seen in the radiotherapy and oncology department, ahmadu bello university teaching hospital, Zaria, Nigeria: 2006–2010	Yes	Yes	Yes	Yes	Yes	100	N/A
Alabi et al., 2010	Clinico-pathological pattern of nasopharyngeal carcinoma in Ilorin, Nigeria	Yes	Yes	Yes	Yes	Yes	100	N/A
Wided et al., 2015	Nasopharyngeal carcinoma incidence in North Tunisia: Negative trends in adults but not adolescents, 1994–2006	Yes	Yes	Yes	Yes	Yes	100	N/A
**Laryngeal hypopharyngeal SCC subgroup**								
Amusa et al., 2011	Laryngeal carcinoma: Experience in Ile-Ife, Nigeria	Yes	Yes	Yes	Yes	Yes	100	N/A
Elfeky et al., 2015	Hypopharyngeal reconstruction: A comparison of three alternatives	Yes	Yes	Yes	Yes	Yes	100	N/A
Iseh, 2011	Total laryngectomy for laryngeal cancer in a Nigerian tertiary health center: Prognosis and outcome	Yes	Yes	Yes	Yes	Yes	100	N/A

HNC, head and neck cancer; SCC, squamous cell carcinoma; NPC, nasopharyngeal carcinoma; N/A, not applicable.
